# New Peptide-Drug Conjugates for Precise Targeting of SORT1-Mediated Vasculogenic Mimicry in the Tumor Microenvironment of TNBC-Derived MDA-MB-231 Breast and Ovarian ES-2 Clear Cell Carcinoma Cells

**DOI:** 10.3389/fonc.2021.760787

**Published:** 2021-10-22

**Authors:** Cyndia Charfi, Michel Demeule, Jean-Christophe Currie, Alain Larocque, Alain Zgheib, Bogdan Alexandru Danalache, Amira Ouanouki, Richard Béliveau, Christian Marsolais, Borhane Annabi

**Affiliations:** ^1^ Theratechnologies Inc., Montréal, QC, Canada; ^2^ Laboratoire d’Oncologie Moléculaire, Département de chimie, Université du Québec à Montréal, Montréal, QC, Canada

**Keywords:** breast cancer, ovarian cancer, Doxorubicin, peptide-drug conjugates, sortilin, vasculogenic mimicry, Docetaxel

## Abstract

Vasculogenic mimicry (VM) is defined as the formation of microvascular channels by genetically deregulated cancer cells and is often associated with high tumor grade and cancer therapy resistance. This microcirculation system, independent of endothelial cells, provides oxygen and nutrients to tumors, and contributes also in part to metastasis. VM has been observed in ovarian cancer and in triple negative breast cancer (TNBC) and shown to correlate with decreased overall cancer patient survival. Thus, strategies designed to inhibit VM may improve cancer patient treatments. In this study, sortilin (SORT1) receptor was detected in *in vitro* 3D capillary-like structures formed by ES-2 ovarian cancer and MDA-MB-231 TNBC-derived cells when grown on Matrigel. *SORT1* gene silencing or antibodies directed against its extracellular domain inhibited capillary-like structure formation. *In vitro*, VM also correlated with increased gene expression of matrix metalloproteinase-9 (MMP-9) and of the cancer stem cell marker CD133. *In vivo* ES-2 xenograft model showed PAS^+^/CD31^-^ VM structures (staining positive for both SORT1 and CD133). TH1904, a Doxorubicin-peptide conjugate that is internalized by SORT1, significantly decreased *in vitro* VM at low nM concentrations. In contrast, VM was unaffected by unconjugated Doxorubicin or Doxil (liposomal Doxorubicin) up to μM concentrations. TH1902, a Docetaxel-peptide conjugate, altered even more efficiently *in vitro* VM at pM concentrations. Overall, current data evidence for the first time that 1) SORT1 itself exerts a crucial role in both ES-2 and MDA-MB-231 VM, and that 2) VM in these cancer cell models can be efficiently inhibited by the peptide-drug conjugates TH1902/TH1904. These new findings also indicate that both peptide-drug conjugates, in addition to their reported cytotoxicity, could possibly inhibit VM in SORT1-positive TNBC and ovarian cancer patients.

## Introduction

Efficient supply of oxygen and nutrients within solid tumors was originally believed to be performed through angiogenesis, an endothelial cell (EC)-mediated process leading to new blood vessels (1). Alternatively, plasticity of aggressive tumors allows an alternate blood perfusion process to take place through an EC-free mechanism termed vasculogenic mimicry (VM) (2–4). Such process further provides a potential dissemination route for highly aggressive and metastatic human cancers, as patterned vessel-like channel structures were reported in melanomas where red blood cells, but not EC, were detected (5, 6). VM is also thought to be associated to cancer therapy resistance, as anti‐angiogenic therapies have shown limited efficacy in the clinical management of metastatic disease (7).

VM has been characterized in carcinomas of ovary, breast, lung, liver, colorectal, prostate, bladder, kidney, sarcomas and gliomas (4, 8), and survival analyses indicate that patients which exhibit high VM processes within their tumors had a poor clinical outcome (9). Meta-analysis studies evaluating the impact of VM on cancer patients survival in 15 types of malignant tumors showed that it was associated with a more aggressive tumor phenotype and a poor 5-year overall survival (8). Recently, cancer stem cells (CSC) and epithelium-to-endothelium transition, a subtype of epithelial-to-mesenchymal transition, have been reported to trigger VM by stimulating cancer cell plasticity and remodeling of the extracellular matrix (ECM) that enabled the connection of VM channels to host blood vessels (10).

Ovarian cancer is among the first carcinomas in which VM was reported to correlate with decreased overall patient survival (8, 11). A retrospective study in 120 ovarian carcinoma samples demonstrated that VM was involved in 43% of all tested tissues (12). In that same study, high expression of the CSC surface marker CD133, was found in 47% of ovarian cancer tissues (12). The presence of both VM and CD133-positive cells was therefore associated with advanced tumor stage, high-grade ovarian carcinoma and non-responsiveness to chemotherapy leading to poor patient prognosis (13). VM has also been reported to be highest in triple negative breast cancer (TNBC) specimens, where CD133-positive cells with CSC characteristics were detected (14). Furthermore, CD133 expression and VM process appear to be closely related in TNBC, as the CSC subpopulation within TNBC-derived MDA-MB-231 cells showed a high degree of plasticity that triggered 3D capillary-like structures formation *in vitro* (15).

Efficient inhibition of cells exhibiting VM is challenging given the combined lack of specific cell surface biomarkers and functional targeting. Recently, a novel targeted therapy strategy was developed against sortilin (SORT1)-positive ovarian and breast cancers (16, 17). As such, tumor suppressive capacities of TH1902, a drug currently tested in a phase 1, open-label first-in-human study in solid cancer (ClinicalTrials.gov Identifier: NCT04706962), were demonstrated against SORT1-positive TNBC xenograft models (17). SORT1 is a key scavenging receptor discovered two decades ago as the first member of the small family of vacuolar protein sorting 10 protein domain (Vps10p) (18). Functional characteristics show that SORT1 has a dual role both in endocytosis and in receptor trafficking allowing the sorting of its ligands from the cell surface to specific subcellular compartments, and the trafficking of pro-neurotrophins such as the neuropeptide neurotensin (NT), proNGF and proBDNF (19–25). It is considered as one of the cells’ own shuttle systems given its role in ligand internalization and cellular trafficking (19). SORT1 is involved in cancer cell proliferation, as well as in cancer cell migration and invasion (26). SORT1 is particularly overexpressed in ovarian cancer as compared to healthy ovarian tissue (27, 28), and is associated with breast cancer invasive phenotype (29). Its expression is elevated in the tumor microenvironment of several other human cancers including prostate, colon, pancreas, skin, and pituitary (30–33).

In the current study, SORT1’s expression and functional role in VM were investigated. SORT1 was detected in 3D capillary-like structures and shown to be essential in VM formation using ES-2 clear cell ovarian cancer and TNBC-derived MDA-MB-231 cell line models. More importantly, conjugation of anticancer drugs, namely Doxorubicin and Docetaxel, to a peptide designed to recognize and to exploit SORT1’s ligand internalization capacity, strongly inhibited VM. Overall, results confirm that these peptide-drug conjugates could efficiently alter the VM process by bringing anticancer drugs into SORT1-positive cancer cells.

## Materials and Methods

### Cells and Reagents

Human ES-2 ovarian cancer cells, as well as TNBC-derived MDA-MB-231 cells were purchased from the American Type Culture Collection (ATCC, Manassas, VA) and cultured for no more than 5 to 10 passages according to the provider’s instructions. Amino acids and resin for the peptide synthesis were from Matrix Innovation Inc (Quebec, QC). Docetaxel was from Wonda Science Inc (Lexington, MA). Doxorubicin-HCl was from Enzo Life Sciences (Farmingdale, NY). The monoclonal anti-SORT1 antibody directed against the 300-422 amino acid sequence within the extracellular domain of SORT1 was from BD Biosciences (612100, San Jose, CA). The polyclonal anti-SORT1 antibody directed against amino acid 800 to the C-terminus of the intracellular domain of SORT1 was from Abcam (ab16640, Cambridge, MA). The respective mouse and rabbit control isotypes IgG1 were from Santa Cruz Biotechnology (Dallas, TX). The anti-mouse-HRP IgG was from Jackson Immuno Research Laboratories (West Grove, PA). All other reagents were from Sigma-Aldrich (Oakville, ON).

### Synthesis of TH19P01

The solid phase peptide synthesis was carried out manually according to the Fmoc strategy using an H-Tyr-2-Cl-Trityl resin. Fmoc amino acids were from Matrix Innovation Inc (Quebec, QC). Fmoc removal was performed using a solution of 20% piperidine in *N,N-*dimethylformamide (DMF) at room temperature for 15 min. Coupling of Fmoc-protected amino acid units was carried out by activation with 2-(6-Chloro-1H-benzotriazole-1-yl)-1,1,3,3-tetramethylaminium hexafluoro-phosphate (HCTU) using *N,N-*diisopropylethylamine (DIPEA) in DMF at room temperature for 40 min. The Fmoc amino acids (2.0 equiv), HCTU (2.0 equiv) and DIPEA (2.5 equiv) were dissolved in DMF and subsequently mixed with the resin manually. Upon completion of synthesis, the peptide resin was subjected to a cleavage cocktail TFA/TIS/H_2_O, (95/2.5/2.5, v/v/v) for 1.5 h. The resin was filtered off and the filtrate was evaporated under reduced pressure. Then, the product was triturated with cold diethyl ether and filtered through a sintered glass funnel to isolate a white solid. The remaining solid was ready for HPLC purification followed by lyophylization. TH19P01 (Ac-GVRAKAGVRN(Nle)FKSESY) was analysed by UPLC/MS and showed a purity >95%, a MW of 1924.2 g/mol, and was used for conjugation with either Doxorubicin or Docetaxel as described below.

### Synthesis of Doxorubicin-TH19P01 Peptide Conjugate (TH1904)

Synthesis, preparation and conjugation of N-Fmoc-DOX-14-O-3,3’-dimethylglutarate was performed, and 3,3’-dimethylglutaric anhydride was used as a linker instead of glutaric anhydride. *TH19P01-(Dmg-FmocDoxo)_2_ conjugate:* DIEA (0.234 mmol) was added dropwise to a solution of DmgOH-FmocDoxo (27.3 mg, 0.03 mmol) and TBTU (9.6 mg, 0.03 mmol) in DMSO (3.0 ml) in order to preactivate the DmgOH-FmocDoxo. The completion of preactivation was monitored by UPLC/MS, a solution of TH19P01 (16 mg, 0.008 mmol) in DMSO (1.0 ml) was then added. The mixture was stirred at room temperature. The reaction was monitored by UPLC/MS until completion. The reaction mixture was then purified using a 30RPC resin column and an AKTA purifier system (10% to 80% ACN; 0.1 Formic acid) to give TH19P01-(DmgOH-FmocDoxo)_2_. *Fmoc deprotection from TH19P01-Dmg-FmocDoxo:* Dmg-FmocDoxo (50 mg) was dissolved in 3.0 ml of DMSO and 10 μl of piperidine was added. The mixture instantaneously turned purple and the removal of the Fmoc group from the Doxorubicin moiety was monitored by UPLC/MS. Deprotection was completed within 10 min. To remove the free Fmoc group and piperidine, the mixture was then loaded directly on a 30RPC resin column for purification using an AKTA purifier system with a gradient of 5-80% ACN; 0.1% Formic Acid. Following lyophilization, TH19P01-(Dmg-Doxo)_2_ (TH1904) was obtained as a reddish powder. Analysis of TH1904 by UPLC/MS showed a purity > 95% and a MW of 3258.6 g/mol.

### Synthesis of Docetaxel-TH19P01 Peptide Conjugate (TH1902)

DIEA (0.21 ml, 1.2 mmol) was added dropwise to a suspension of Docetaxel (0.81 g, 1.0 mmol) and succinic anhydride (105 mg, 1.05 mmol) in DMSO (5 ml) under stirring. The mixture was stirred at room temperature and monitored by UPLC-MS. After 2 h, the reaction was completed. The solvent was removed, and the resulting residue was dissolved in dichloromethane and loaded on Biotage silica column for purification. DoceSuOH was obtained as a white powder after lyophilization, and purity > 95% assessed by UPLC/MS. DIEA (0.234 mmol) was added dropwise to a solution of DoceSuOH (213 mg, 0.234 mmol) and TBTU (75 mg, 0.234 mmol) in DMSO (3-4 ml) in order to preactivate the DoceSuOH. The completion of preactivation was monitored by UPLC/MS, and then a solution of TH19P01 (120 mg, 0.062 mmol) in DMSO (0.2 ml) was added. The mixture was stirred at room temperature. The reaction was monitored by UPLC/MS until completion. The reaction mixture was purified using a 30RPC resin column and an AKTA purifier system (10% to 80% ACN) to give TH19P01-(SuDoce)_2_ or TH1902 as a white powder after lyophilisation. Purity of TH1902 was evaluated by UPLC/MS and was > 95%.

### Western Blotting

ES-2 ovarian cancer cells and TNBC-derived MDA-MB-231 cells were homogenized in lysis buffer (150 mM NaCl, 10 mM Tris–HCl, pH 7.4, 1 mM EDTA, 1 mM ethyleneglycol-O, O’-bis(2-aminoethyl)-N, N, N’, N’-tetraacetic acid (EGTA), 0.5% (vol/vol) Nonidet P-40 and 1% (vol/vol) Triton X-100) supplemented with a complete protease inhibitor cocktail from Calbiochem (San Diego, CA). Cells were incubated for 30 min at 4°C, sonicated and centrifuged at 15,000g for 10 min at 4°C. Equal amounts of protein (20 ug) were separated by SDS–polyacrylamide gel electrophoresis (PAGE). Proteins were then electrotransferred to a polyvinylidene fluoride (PVDF) membrane and blocked for 1 h at room temperature using 5% non-fat dry milk in Tris-buffered saline (150 mM NaCl, 20 mM Tris–HCl, pH 7.5) containing 0.1% Tween-20 (TBST). Membranes were washed in TBST and incubated overnight with primary antibodies against SORT1 (1/1,000 dilution) or GAPDH (1/30,000 dilution) diluted in TBST containing 3% BSA and 0.05% NaN_3_. Membranes were washed in TBST and incubated for 1 h at room temperature with horseradish peroxidase-conjugated anti-mouse or anti-rabbit IgG (1/5000 dilution) in TBST containing 5% non-fat dry milk. Membranes were washed again in TBST and signals were detected using chemiluminescence (Amersham Biosciences, Baie d’Urfé, QC).

### Immunofluorescent Staining

For SORT1 staining, cells were seeded in 8-chambers Ibidi dishes (Ibidi) containing 50 uL Matrigel, and incubated for 24 hours. Cells were then washed in PBS, fixed for 15 min in 2% paraformaldehyde (PFA), permeabilized with 1% Triton X-100 in PBS for 5 min, and finally washed in PBS. Cells were blocked in PBS containing 10% normal host serum and 0.05% Triton, and incubated overnight with an anti-SORT1 primary antibody (Abcam, 1/100). Cells were washed with PBS and incubated for 1 hour with Alexa-Fluor^488^-conjugated secondary antibody (1/1,000; Invitrogen), washed with PBS, stained with 2 µg/ml DAPI (Invitrogen) for 5 min, washed again in PBS and mounted onto slides using Prolong Gold antifade reagent. Cells were finally digitalized by confocal microscopy (Nikon A1) and analyzed using the NIH ImageJ Version 1.4.21 software.

### SORT1 RNA Interference

ES-2 or MDA-MB-231 cells were transiently-transfected for 24 h with 100 nM of a scrambled sequence (AllStar Negative Control siRNA, 1027281) or a human siRNA against *SORT1* (Hs_SORT_5 FlexiTube siRNA: SI03115168; Qiagen, Valencia, CA) using Lipofectamine 2000 (ThermoFisher Scientific). Extent of *SORT1* gene silencing was assessed by RT-qPCR and, at the protein level, by Western blotting as described above.

### Total RNA Isolation, cDNA Synthesis and Real-Time Quantitative PCR

Total RNA was extracted from ES-2 and MDA-MB-231 cells cultured on top of Matrigel using TriZol reagent (Life Technologies, Gaithersburg, MD). For cDNA synthesis, 2 μg of total RNA were reverse-transcribed using a high capacity cDNA reverse transcription kit (Applied Biosystems, Foster City, CA). cDNA was stored at -80°C prior to PCR. Gene expression was quantified by real-time quantitative PCR using iQ SYBR Green Supermix (Bio-Rad, Hercules, CA). DNA amplification was carried out using an Icycler iQ5 (Bio-Rad, Hercules, CA) and product detection was performed by measuring binding of the fluorescent dye SYBR Green I to double-stranded DNA. The QuantiTect primer sets were from Qiagen: CD133 (QT00075586), MMP-9 (QT00040040), NTSR3/Sortilin (QT00073318), PPIA (QT01866137), and GAPDH (QT00079247). The relative quantities of target gene mRNA were compared against two internal controls, GAPDH and PPIA mRNA, and were measured by following a ΔCT method employing an amplification plot (fluorescence signal *vs*. cycle number). The difference (ΔCT) between the mean values in the triplicate samples of target gene and those of GAPDH and PPIA mRNA were calculated by iQ5 Optical System Software version 2.0 (Bio-Rad, Hercules, CA). The relative quantified value (RQV) was expressed as 2^-ΔC^T. Vehicle (0.1% DMSO) was kept constant in all treatments.

### *In Vitro* Vasculogenic Mimicry Assay

VM was assessed *in vitro* using Matrigel to monitor 3D capillary-like structures formation [26]. In brief, each well of a 96-well plate was pre-coated with 50 μl of Matrigel. ES-2 or MDA-MB-231 cell suspension in culture media (1.8×10^4^ cells/200 μl) was then seeded on top of gelified Matrigel. Tested compounds were added to the cell culture media and incubated at 37°C in a CO_2_ incubator. Pictures were taken over time using a digital camera coupled to a phase-contrast inverted microscope. The number of loops (blue) and area covered upon tube branchings (red) formed by the cells were quantified using either the Wimasis Analysis software (Cordoba, Spain) or the ImageJ Software.

### Tumor Xenografts

Animals were obtained from Charles River Laboratories, Inc. (St-Constant, QC) and allowed to acclimate for 5 days before experiments. Female CD-1 nude mice (Crl : NU-Foxn1nu; 20-25 g, 4-6 weeks old) were used for xenograft tumor models. Tumor xenografts were established by subcutaneous inoculation of 7x10^6^ ES-2 ovarian cancer cells, resuspended in 100 μL of HBSS and injected into the right flank of CD-1 nude mice under light isoflurane anesthesia. Palpable tumors typically developed within 7-10 days. When palpable tumors reached a volume of ~100 mm^3^, tumors were then evaluated three times a week by two-dimensional measurements taken with an electronic caliper. Tumor volume was calculated according to the following formula: tumor volume (mm^3^) = π/6 x length x width^2^. Tumors were collected when they reached approximately 1500 mm^3^ in size. All mice were maintained in a pathogen-free environment. All animals used were handled and maintained in accordance with the Guidelines of the Canadian Council on Animal Care. Animal protocols were approved by the Institutional Animal Care and Use Committee of Université du Québec à Montréal.

### Immunohistochemistry

For detection of VM-associated structures, formalin-fixed, paraffin-embedded tumor tissue sections were stained with primary antibodies against mouse CD31 (#CM303A, Biocare Medical, Pacheco, CA), human CD133 (#130-090-422, Miltenyi Biotec, San Diego, CA) and human sortilin (#MABN1792, EMD Millipore Corporation, Temecula, CA). Then, the sections were treated with 0.5% periodic acid-Schiff (PAS) solution for 15 min. After rinsing with distilled water for 2 min, tumor tissue sections were placed into Schiff solution for 15-30 min in a dark chamber and rinsed with distilled water for three times. Afterwards, sections were counterstained with hematoxylin. 3D capillary-like structures were found to be formed by CD31-negative cancer cells in hematoxylin-eosin stained slides, and VM validated by CD31/PAS double-staining and further identified by the detection of PAS-positive loops surrounded with tumor cells (not EC). For each tumor, one single staining (sortilin, CD31, CD133 or PAS) or one co-staining (sortilin-PAS, mouse CD31-PAS or CD133-PAS) was performed. With respect to immunohistochemical staining for CD133 and sortilin, formalin-fixed, and paraffin-embedded slides from cancer tissue sections were deparaffinized using proprietary dewax reagents in xylene and then rehydrated with decreasing concentrations of ethanol followed by an endogenous peroxidase block. Then, antigen retrieval was performed by heat-induced epitope retrieval (HIER) techniques with ER1 solution (Lecia) for 30 min at 100°C (CD133 and sortilin) or digested with trypsin enzyme for 5 minutes at 37°C (CD31). Afterwards, slides were incubated with 3% H_2_O_2_ for blocking the endogenous peroxidase, and then with 20% goat serum for reducing nonspecific binding. Mouse anti-human CD133 monoclonal antibody (1/50 dilution) was added to the slides after washing with phosphate buffered saline, and slides incubated at 4°C overnight. On the next day, slides were incubated in a peroxidase-conjugated rabbit anti mouse secondary antibody (1/200 dilution) (Dako Cytomation, Carpinteria, CA) for 30 min. Diaminobenzidine was used for visualizing the reactions. Finally, the slides were counterstained with Leica proprietary hematoxylin and mounted for analysis. Images were captured using Nanozoomer slide scanner (Hamamatsu Photonics K.K., Hamamatsu, Japan) and analyzed with Aperio ImageScope (Leica Biosystems, version 12.4.3.5008, Buffalo Grove, Il). The CD133-positive slides were set as positive controls, while slides incubated with tris-buffered saline instead of monoclonal antibody were set as negative controls. CD133-stained cells were counted in at least nine random and non-overlapping fields at 400Å~ magnification. For detection of CD133-positive tumor cells, staining degree was scored and the presence of either membrane and/or cytoplasmic staining was considered a positive signal. For statistical analysis, patients were then defined as CD133-negative (0% CD133-positive tumor cells) and CD133-positive (>0% CD133-positive tumor cells).

### Real-Time Cell Migration Assay

Cell migration experiments were carried out using the Real-Time Cell Analyser Dual-Plate xCELLigence system according to the supplier’s instructions (Roche Diagnostics, Laval, QC). Transfected siScrambled or siSORT1 cells (25,000 cells/well) were seeded in serum-free medium onto a CIM-Plates 16 (Roche Diagnostics). These plates are similar to conventional Transwells (8-μm pore size) but with gold electrode arrays at the bottom side of the membrane, which provide measurement of cell migration in real-time. Prior to cell seeding, the underside of the wells from the upper chamber was coated with 25 μL of 0.15% gelatin in PBS for 1 h at 37°C. The lower chamber was filled with serum-free medium. The upper chamber of each well was filled with 100 μL of ES-2 and MDA-MB-231 cells (2.5x10^5^ cells/mL) pre-treated for 2 h with or without 2 µM of either TH1902 or TH1904. After 30 min of adhesion, cell migration was next monitored every 5 min to 12 h. The impedance value measured was then expressed as an arbitrary unit termed the “Cell Index” which reflects the number of migration-active cells.

### Statistical Data Analysis

Data are expressed as means ± standard deviation (SD). Statistical analysis was done using t-test for comparing two samples, while analysis by one-way ANOVA followed by Dunnett’s multiple comparisons test for 3 or more samples. A value of p < 0.05 was considered significant and an asterisk (*) identifies such significance in the figures.

## Results

### SORT1 Is Expressed at the Surface of Ovarian and TNBC Cancer Cells Forming *In Vitro* 3D Capillary-Like Structures

**Figure 1:**
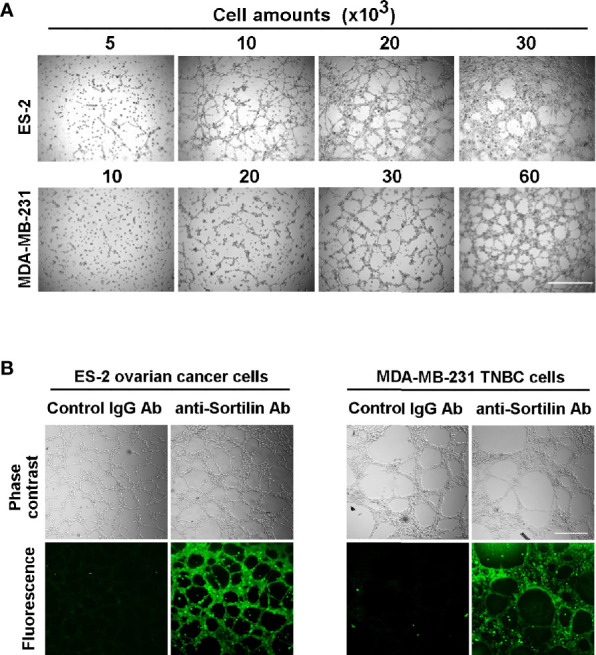
*In vitro* vasculogenic mimicry by ES-2 ovarian cancer cells and by TNBC-derived MDA-MB-231 cells. **(A)** Increasing amounts of ES-2 and MDA-MB-231 cancer cells were seeded ontop of Matrigel and left to form 3D capillary-like structures for 24 h as described in the Methods section. Scale bar = 1000 μm **(B)** Immunolabeling of sortilin in 3D capillary-like structures formed by ES-2 or MDA-MB-231 cancer cells was performed using confocal fluorescent microscopy. Control stainings were performed with only the secondary anti-rabbit antibody. Representative 3D structure stainings are shown for each cell model. Scale bar = 100 μm.

In order to identify the cell models and optimize the conditions that lead to *in vitro* VM, increasing amounts of ES-2 ovarian cancer and TNBC-derived MDA-MB-231 cells were seeded ontop of Matrigel. Mature 3D capillary-like structures were formed at 30x10^3^ and 60x10^3^ cells for ES-2 and MDA-MB-231 cells, respectively (**Figure 1A**). Whether SORT1 was expressed in cells forming these 3D structures was next investigated. Results show that SORT1 was detected in ES-2 and MDA-MB-231 3D capillary-like structures as assessed by fluorescent confocal microscopy (**Figure 1B**). These results indicate that SORT1 is present in ES-2 and MDA-MB-231 cancer cells that form *in vitro* VM.

### *CD133* and *MMP-9* Gene Expression Increases With 3D Capillary-Like Structures Maturation

**Figure 2:**
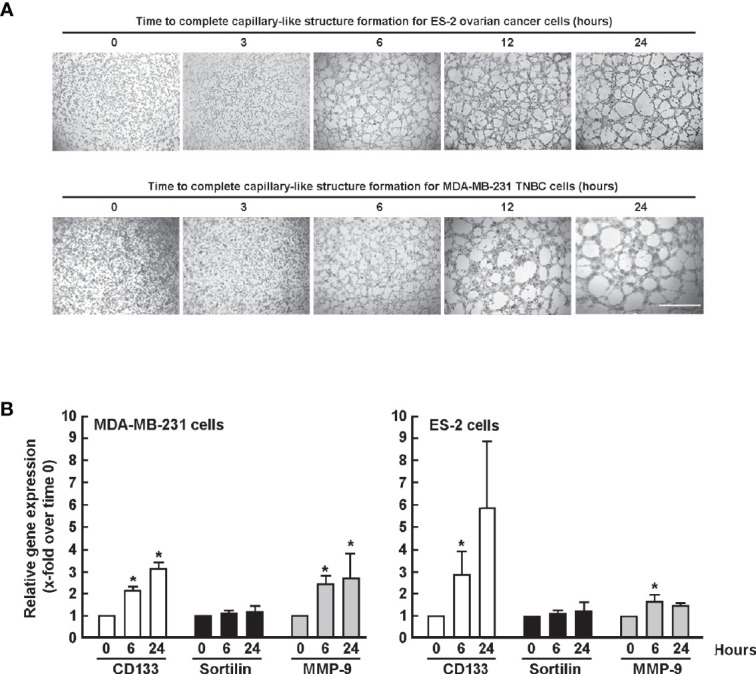
Gene expression of *SORT1*, *CD133* and *MMP-9* during *in vitro* vasculogenic mimicry. **(A)** ES-2 ovarian cancer and MDA-MB-231 TNBC-derived cells were seeded ontop of Matrigel and *in vitro* VM assessed for up to 24 h. Scale bar = 1000 μm. **(B)** Total RNA was isolated from ES-2 and MDA-MB-231 cells at initial (t = 0 h), at early maturation (t = 6 h) and complete maturation (t = 24 h) of 3D capillary-like structures. Gene expression levels of *SORT1*, *CD133*, and *MMP-9* were assessed in triplicate by RT-qPCR from three independent experiments as described in the Methods sections.

The expression of SORT1, matrix metalloproteinase (MMP)-9, and of CD133 CSC biomarker during *in vitro* VM was next assessed. ES-2 and TNBC-derived MDA-MB-231 cells formed 3D structures starting around 3-6 h upon seeding on Matrigel (**Figure 2A**). Cells were harvested at initial (t = 0 h), mid-maturation (t = 6 h), and complete maturation (t = 24 h) time points, total RNA isolated, and gene expression levels assessed by RT-qPCR as described in the Methods section. During 3D capillary-like structure formation, in both cell line models tested, whereas *SORT1* expression remained unchanged, that of *CD133* and *MMP-9* increased with structure maturation in MDA-MB-231. *CD133* expression also increased in ES-2 cells, but that of *MMP-9* remained mostly uninduced (**Figure 2B**). This suggests that a common CD133-positif CSC phenotype correlates with the maturation of capillary-like structures, and that concomitant increase in MMP-9 may contribute to VM regulation within specific cancer cell models.

### SORT1 Is Expressed in Vasculogenic Mimicry Structures From Tumor Xenografts

**Figure 3:**
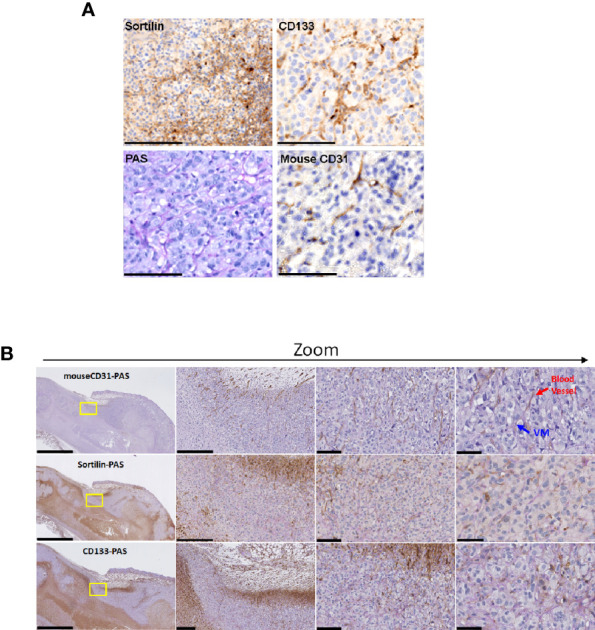
SORT1 and CD133 expression in ES-2 ovarian tumor xenografts. Tumor xenografts were established by subcutaneous inoculation of 7x10^6^ ES-2 ovarian cancer cells, resuspended in 100 μL of HBSS and injected in the right flank of CD-1 nude mice. Tumors were collected when they reached 1500 mm^3^. **(A)** Sortilin, CD133, CD31 and PAS stainings were performed as described in the Methods section. **(B)** Co-staining with PAS was also performed for CD31 (CD31-PAS), sortilin (Sortilin-PAS) and CD133 (CD133-PAS). VM structures (blue arrows) and blood vessels (red arrows) are indicated. Scale bars represent 2500, 250, 100 and 50 µm respectively (left to right column). Representative images are shown from two different ES-2 xenograft tumors.

To further evaluate the contribution of SORT1 in ovarian tumor tissues, ES-2 xenograft tumor models were generated as described in the Methods section, and immunohistochemistry was performed in tumor tissue sections with specific primary antibodies against SORT1, CD31, and CD133. Sections were then co-stained with either PAS-anti-CD31, PAS-anti-SORT1 or PAS-anti-CD133 (**Figure 3A**), and counterstained with hematoxylin-eosin. As previously validated, the VM structures observed were PAS positive and CD31 negative (34) (**Figure 3B**, blue arrows), whereas blood vessels stained positive for CD31 (**Figure 3B**, red arrows). SORT1 and CD133 stainings were both associated with cancer cells and in part with PAS staining. Overall, these results indicate that both SORT1 and CD133 are present in VM structures formed within ES-2 tumor xenografts.

### Functional SORT1 Is Required for *In Vitro* Vasculogenic Mimicry

**Figure 4:**
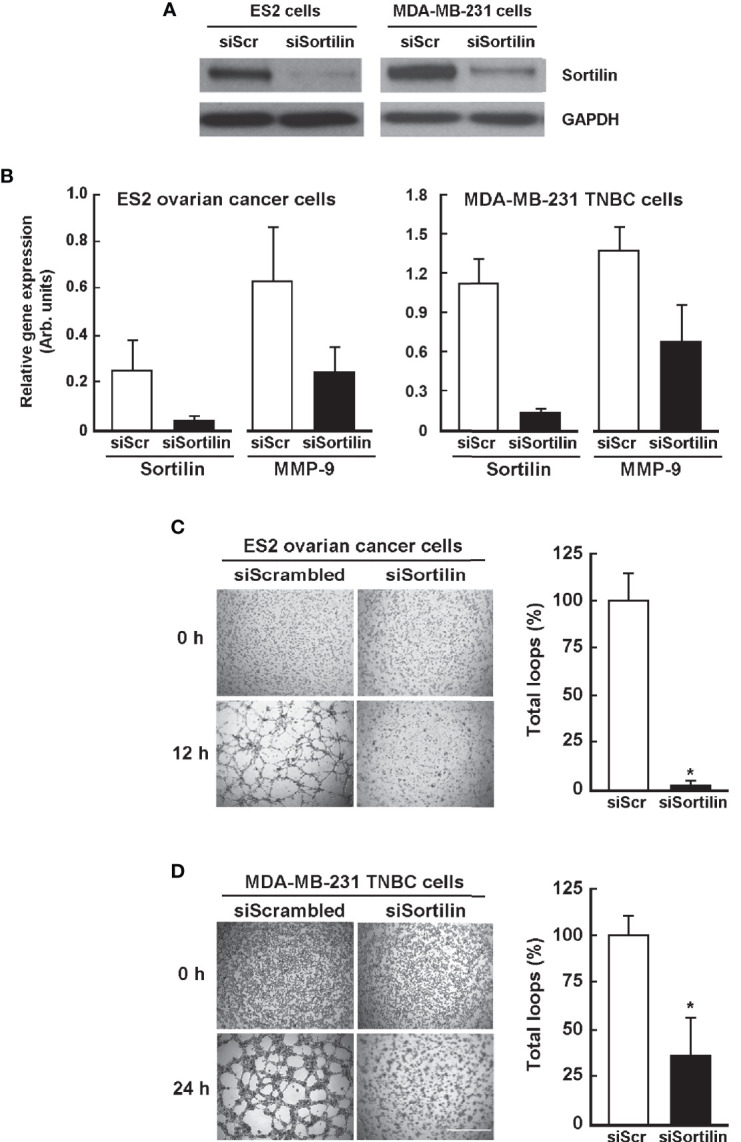
Effect of sortilin gene silencing on *in vitro* vasculogenic mimicry. Cells were transiently transfected with either scrambled siRNA (siScr) or specific sortilin siRNA (siSortilin) as described in the Methods section. The extent of sortilin silencing was assessed in ES-2 and MDA-MB-231 cells at **(A)** the protein level by immunoblotting of cell lysates, and at **(B)** the gene expression levels by RT-qPCR from total RNA. MMP-9 gene expression was also assessed in both cell models transfected with either siScr or specific siSortilin. Transfected cells were then seeded ontop of Matrigel for 12 h (ES-2 cells), or 24 h (MDA-MB-231 cells). Quantitation of total number of loops was performed for **(C)** ES-2 cells and **(D)** MDA-MB-231 cells as described in the Methods section. Quantitation performed in **(A–D)** results from 3 independent experiments. Scale bar = 1000 μm.

The contribution of SORT1 in the formation of 3D capillary-like structures by ES-2 ovarian cancer cells and MDA-MB-231 TNBC cells was next assessed. When SORT1 expression was repressed through specific siRNA-mediated gene silencing (**Figure 4A**), MMP-9 was also decreased (**Figure 4B**). SORT1 silencing lead to decreased 3D structures when compared to scrambled siRNA in ES-2 ovarian cancer cells (**Figure 4C**) and in MDA-MB-231 TNBC cells (**Figure 4D**). SORT1 silencing did not affect cell viability (data not shown), and this is in agreement with previous studies also showing that this did not affect cell proliferation as well (29). Quantitative analysis of the total loops formed by the cells was performed and results indicate that the number of loops was decreased by 60-90% in both cell models upon SORT1 silencing. These data confirm that SORT1 is essential for the formation of 3D capillary-like structures by MDA-MB-231 and ES-2 cells, and that a transcriptional regulation axis linking SORT1 to MMP-9, in part, explains the decreased VM.

### TH1902 and TH1904 Impact on Vasculogenic Mimicry

**Figure 5:**
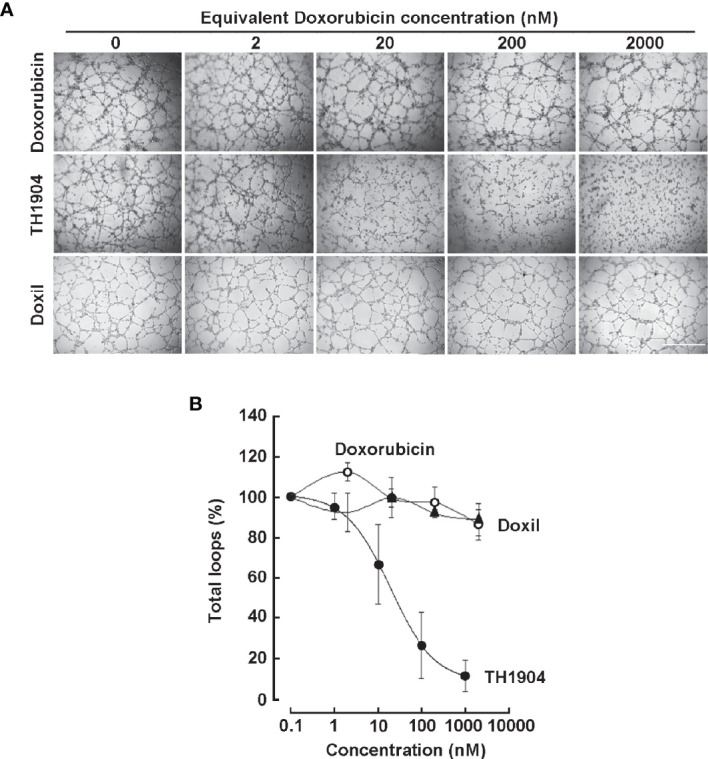
Effect of TH1904, Doxorubcin and liposomal Doxorubicin (Doxil) on *in vitro* vasculogenic mimicry. **(A)** Representative pictures of 3D capillary-like structures formed at 12 h from ES-2 ovarian cancer cells seeded ontop of Matrigel in the presence of increasing concentrations of TH1904, Doxorubicin, or Doxil. Control (vehicle) was 0.1% DMSO and was kept constant through out the range of cencentrations tested. Scale bar = 1000 μm. **(B)** 3D capillary-like structures were quantified for total loop numbers as described in the Methods section. Results represent the mean ± SD for n=2 (Doxil, Doxorubicin) and n=4 (TH1904).

**Figure 6:**
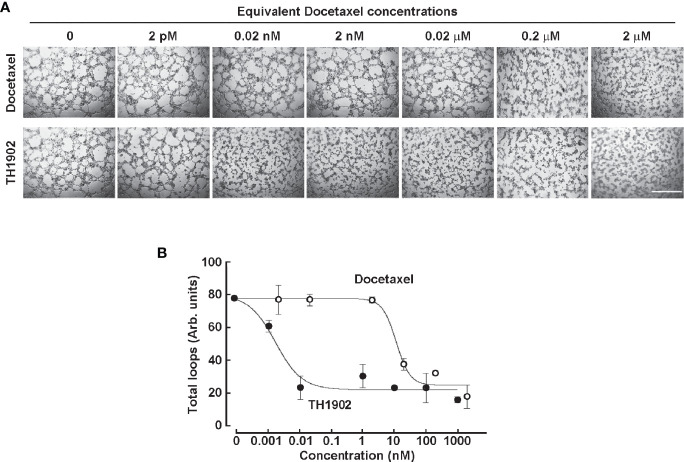
Effect of TH1902 and Docetaxel on *in vitro* vasculogenic mimicry. **(A)** Representative pictures of 3D capillary-like structures fromed at 24 h from TNBC-derived MDA-MB-231 cells seeded ontop of Matrigel in the presence of increasing concentrations of either Docetaxel or TH1902. Scale bar = 1000 μm. **(B)** 3D capillary-like structures were quantified at 24 h for total loop numbers as described in the Methods section. Results represent the mean ± SD for n=3 (Docetaxel, TH1902).

The SORT1 receptor-mediated internalization and impact of TH1902 and TH1904 conjugates on VM were next investigated. 3D capillary-like structures formation by ES-2 ovarian cancer cells was tested in the presence or not of unconjugated Doxorubicin, liposomal Doxorubicin (Doxil), or TH1904 (**Figure 5A**). After 12 h, increasing concentrations of TH1904 strongly inhibited *in vitro* VM whereas unconjugated Doxorubicin or Doxil, up to 20 µM, slightly affected VM (less than 10%) (**Figure 5B**). TH1904 IC_50_ value was 54 nM for the number of loops inhibition (**Figure 5B**). Proof-of-concept supporting SORT1-mediated inhibition of VM was also provided with a Docetaxel conjugate (TH1902). Stronger inhibition of VM, as compared to Docetaxel alone, was observed (**Figure 6A**). The TH1902 IC_50_ value for the number of total loops was ~30 pM as compared to 10 nM for unconjugated Docetaxel (**Figure 6B**). The unconjugated TH19P01 peptide itself was found not to alter VM (data not shown).

### TH1902 and TH1904 Require SORT1 to Inhibit Cancer Cell Migration

**Figure 7:**
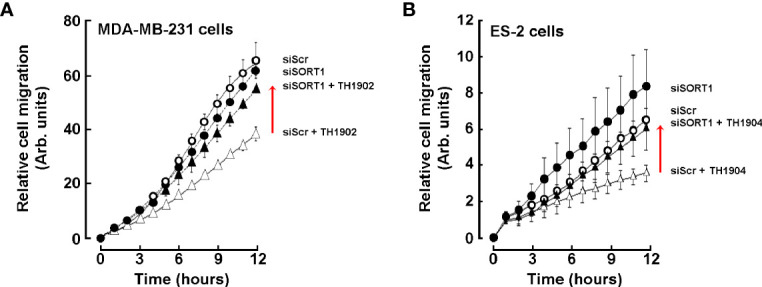
Inhibition of MDA-MB-231 and ES-2 cancer cell migration by TH1902 and TH1904. MDA-MB-231 and ES-2 cancer cells were transiently transfected with either scrambled siRNA (siScr) or specific SORT1 siRNA (siSORT1) as described in the Methods section. **(A)** Transfected siScr (open circles) or siSORT1 (closed circles) MDA-MB-231 TNBC cells were then pre-incubated for 2 h with vehicle or with 2 µM of TH1902 respectively in siScr (open triangels) or siSORT1 (closed triangles). **(B)** Control siScr (open circles) or siSORT1 (closed circles) ES-2 cells were then pre-incubated with vehicle or for 2 h with 2 µM of TH1904 respectively in siScr (open triangles) or siSORT1 (closed triangles). Cells in **(A, B)** were harvested and metastatic potential assessed by following migration in real-time as described in the Methods section. Results are expressed as relative to initial time-point measurements. Data represent the mean ± SEM of two independent experiments, performed in duplicate wells.

Cell migration is among the multiple events required by cancer cells to trigger VM. It previously was shown that the VM capacity of cancer cells was strongly associated with an invasive phenotype, in part determined by their cell migration capability (35). Therefore, whether the inhibition of VM by TH1902 and TH1904 was associated with suppression of such invasive phenotype was investigated. Real-time cell migration was assessed in ES-2 and MDA-MB-231 cells as described in the Methods section. Pretreatment of MDA-MB-231 cells (**Figure 7A**) or ES-2 cells (**Figure 7B**) cells with TH1902 or TH1904 respectively inhibited cell migration. More importantly, specific silencing of *SORT1* significantly prevented the anti-migratory effect of both conjugates. It is to note that, in our experimental settings, SORT1 silencing did not alter basal cell migration as specifically assessed through impedance values collected through the xCELLigence real-time cell migration assay. Overall, these results indicate that both conjugates alter cell migration in either TNBC or ovarian cancer cell models through a SORT1-dependent mechanism. The effects of TH1902 and TH1904 on cell migration further support the molecular rationale that inhibition of VM activity may result, in part, from the reduction of cancer cell invasive phenotype.

### Functional Evidence for SORT1 Contribution in Vasculogenic Mimicry

**Figure 8:**
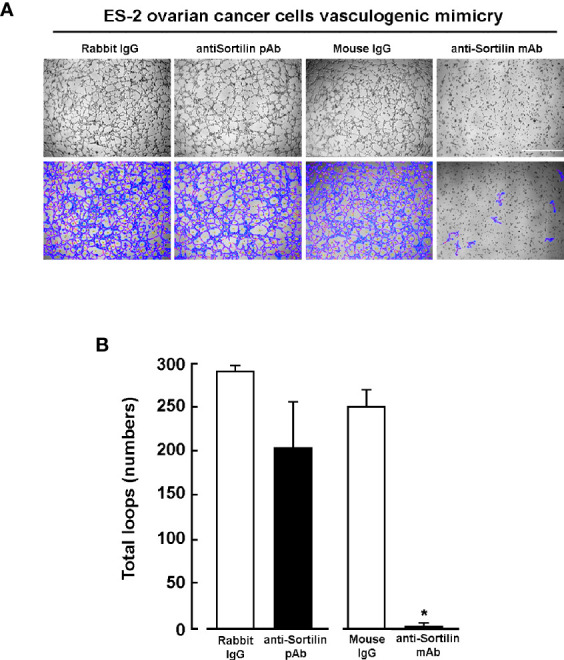
Inhibition of ES-2 ovarian cancer cells *in vitro* vasculogenic mimicry by an anti-sortilin antibody directed against the sortilin extracellular domain. **(A)** ES-2 ovarian cancer cells were seeded ontop of Matrigel in the presence of 25 nM of either mouse IgG1, rabbit IgG, anti-SORT1 mouse mAb, or anti-SORT1 rabbit pAb. Pictures were taken after 12 h of 3D capillary-like structures formation. The number of loops (blue) and area covered upon tube branchings (red) formed by the cells were quantified as described in the Methods section. Scale bar = 1000 μm. **(B)** Quantification of total loop numbers. (n=3 ± SD).

**Figure 9:**
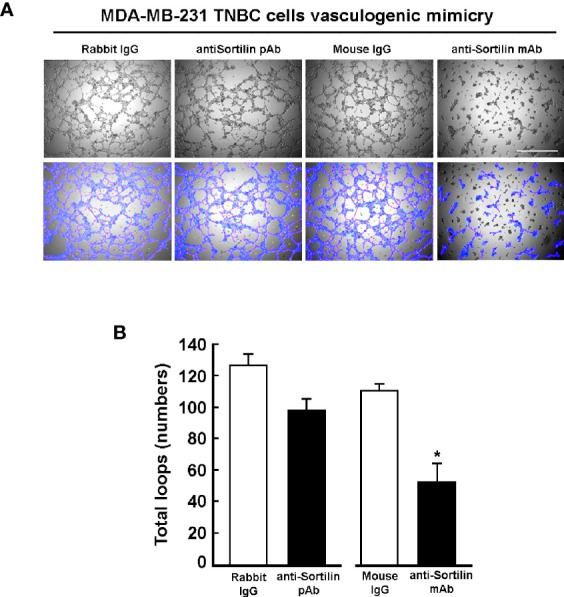
Inhibition of TNBC-derived MDA-MB-231 cells *in vitro* vasculogenic mimicry by an anti-SORT1 antibody directed against the SORT1 extracellular domain. **(A)** MDA-MB-231 cells were seeded ontop of Matrigel in the presence of 25 nM of either mouse IgG1, rabbit IgG, anti-SORT1 mouse mAb, or anti-SORT1 rabbit pAb. Pictures were taken after 24 h of 3D capillary-like structures formation. The number of loops (blue) and branching (red) formed by the cells were quantified as described in the Methods section. Scale bar = 1000 μm. **(B)** Quantification of total number of loops. (n=3 ± SD).

To further examine the functional role of SORT1 in VM, antibodies directed against either the intracellular or the extracellular domains of SORT1 were tested. ES-2 ovarian cancer cells (**Figure 8**) or MDA-MB-231 TNBC cells (**Figure 9**) were seeded ontop of Matrigel in the presence of either a non-specific mouse IgG, a polyclonal anti-SORT1 antibody, which recognizes an intracellular C-terminal domain of SORT1, or a monoclonal anti-SORT1 antibody directed against the extracellular domain of SORT1 (**Figure 8A**). Results show that the monoclonal anti-SORT1 antibody inhibited the formation of 3D capillary-like structures in both ES-2 ovarian cancer (**Figure 8B**) and MDA-MB-231 TNBC cells (**Figure 9B**), whereas the non-specific IgG tested were inefficient to do so. The VM inhibition observed with the antibodies and upon SORT1 silencing suggests that specific domain recognized by the antibody is important for the role of SORT1 in VM formation.

## Discussion

Angiogenesis was initially considered as the only mechanism supporting tumor blood supply. Anti-angiogenic therapies have thus accordingly been designed to target vascular EC and to inhibit the formation of tumor blood vessels (1). While numerous preclinical models have recognized the efficient use of angiogenesis inhibitors to limit tumor growth, only a growth delay has unfortunately been achieved in patients (2). This has been attributed to the fact that tumor vasculature is more complex than first expected and that alternative mechanisms for re-vascularization take place and often lead to chemotherapy resistance. VM represents one of these alternative mechanisms, associated with a poor prognosis factor in many malignancies, and considered as a potential mechanism of cancer resistance to anti-angiogenic drugs (8). The present study is the first to report that SORT1 is a key player involved in VM formation, and that specific strategies exploiting SORT1-mediated peptide-drug conjugates internalization process results in very efficient inhibition of *in vitro* VM in the SORT1-positive ovarian and TNBC cell models tested herein.

*In vitro* tube formation assays using Matrigel demonstrate that both EC and highly invasive cancer cells can form 3D capillary-like structures (35). During VM, remodeling of the extracellular matrix through the hydrolytic activities of MMP, among other proteases, and migration to organize into 3D capillary-like structures, are prerequisite steps of this process recapitulating mechanisms involved in cancer promotion. Interestingly, recruitment of CD133-positive cells with CSC characteristics has been associated with VM in TNBC and ovarian cancer (12, 14). In the present study, increases in both CD133 and MMP-9 expression was observed during VM. Repressing SORT1 expression/function, through siRNA-mediated gene silencing or through the use of specific anti-SORT1 blocking antibody, demonstrates that the modulation of its expression (siRNA) or function (blocking Ab) clearly impacts on the capacity of cells to perform vasculogenic mimicry. The mechanistic aspect being, in part, explained herein through the consequent downregulation of MMP-9 expression which matrix metalloproteinase activity is required to generate appropriate interaction with the ECM proteins and to lead to 3D-capillary-like structures *in vitro*.

Various studies have reported that SORT1 expression levels are associated with different types of cancers and that SORT1 could play a role in tumorigenesis (27–30). Recently, SORT1 has been shown to play a new role in both the assembly of a tyrosine kinase complex and in exosome release (19, 36). This novel complex, termed TES complex, is present in exosomes and results in the linkage of two tyrosine kinase receptors, TrkB and EGFR, with SORT1. Interestingly, the TES complex conveys a control on the tumor microenvironment and initiates the activation of angiogenesis *via* exosome transfer. Therefore, it is inferred that SORT1 and its partners may exert paracrine regulation through exosome transfer and control of the tumor microenvironment. SORT1-positive extracellular vesicules have in fact been detected in our cells by immunofluorescence using anti-SORT1 Ab (not shown) and would understandably require further heavy investigation to potentially complement the current study. Here, data obtained, using siRNA gene silencing and anti-SORT1 antibodies directed against its extracellular domain, indicate that SORT1 receptor is a key element in VM, and that its targeting could therefore be of clinical relevance (37).

To the best of our knowledge, this is the first evidence that SORT1-positive cancer cells can form VM while the SORT1-depleted cancer cells cannot. In fact and although this appears to be cell specific, SORT1 silencing was reported to decrease focal adhesion kinase (FAK) phosphorylation and to correlate with reduced breast (29) and pancreatic cancer cell invasion (36). More importantly, pharmacological evidence leading to VM inhibition was found to occur through the FAK/AKT signaling pathway (37, 38). Intriguingly, SORT1 silencing was unable to reduce Src phosphorylation in PANC-1 cells (37), whereas it did in MDA-MB-231 cells (29). This leads to the conclusion that the SORT1 signaling axis could appear to be cell specific, non-universal, and more complex than originally assumed with several stages of upstream phosphorylation cascades involved. The SORT1/FAK signaling crosstalk, in part involving MMP-2 and MMP-9 (39), may so far molecularly integrate the combined impact of SORT1 targeting and of SORT1-mediated internalization of the peptide-drug conjugates tested herein. These evidences clearly demonstrate that functional SORT1 is essential for ES-2 and MDA-MB-231 cancer cells ability to trigger VM and that this required its extracellular domain. *In vivo*, PAS positivity and negative CD31 staining are often considered the standard to quantify VM (40, 41). Here, conclusive *in vivo* evidence is are presented showing that VM tubular structures, positively stained for PAS and negatively stained for CD31, are present in ES-2 ovarian tumor xenografts. Furthermore, these VM structures were also found positive for SORT1 and CSC biomarker CD133 stainings.

Although research efforts have increased in the development of VM targeted strategies, only few agents have been shown to inhibit VM. Thalidomide has been found to decrease VM in B16F10 melanoma by reducing expression of NF-κB, VEGF, MMP-2, MMP-9, and of proliferating cell nuclear antigen (42). Genistein was found to reduce VM in melanoma by down-regulating vascular endothelial (VE)-cadherin (43), isoxanthohumol reduced VM in breast cancer cells (44), curcumin has been shown to inhibit VM in squamous cell carcinoma of the larynx, hepatocellular carcinoma and murine choroidal melanoma (45–48), and green tea-derived catechins inhibited *in vitro* VM in ovarian and prostate cancer cell models (49, 50). However, the use of curcumin and green tea catechins in therapy suffers major drawbacks including their poor absorption, fast metabolism, quick systemic elimination, low bioavailability, poor pharmacokinetics, low stability, and low penetration targeting efficacy (51, 52). Such limitations in future therapeutic applications may efficiently be circumvented through the current peptide-drug conjugation for precise targeting strategies described herein. Here, results further clearly indicate that exploiting SORT1’s peptide internalizing functions with two new peptide-drug conjugation chemistry, TH1902 and TH1904, or that interfering with crucial extracellular domains of SORT1 all lead to efficient *in vitro* VM inhibition. Moreover, a transcriptional regulation axis linking SORT1 to MMP-9 suggests that inhibiting SORT1-dependent VM activity may concomitantly be accompanied by a reduction in cancer cell invasiveness. Overall, these results suggest that the TH1902 and TH1904 peptide-drug conjugates have a dual anticancer activity by targeting both cell migration and VM in SORT1-positive cancers.

## Conclusion

**Figure 10:**
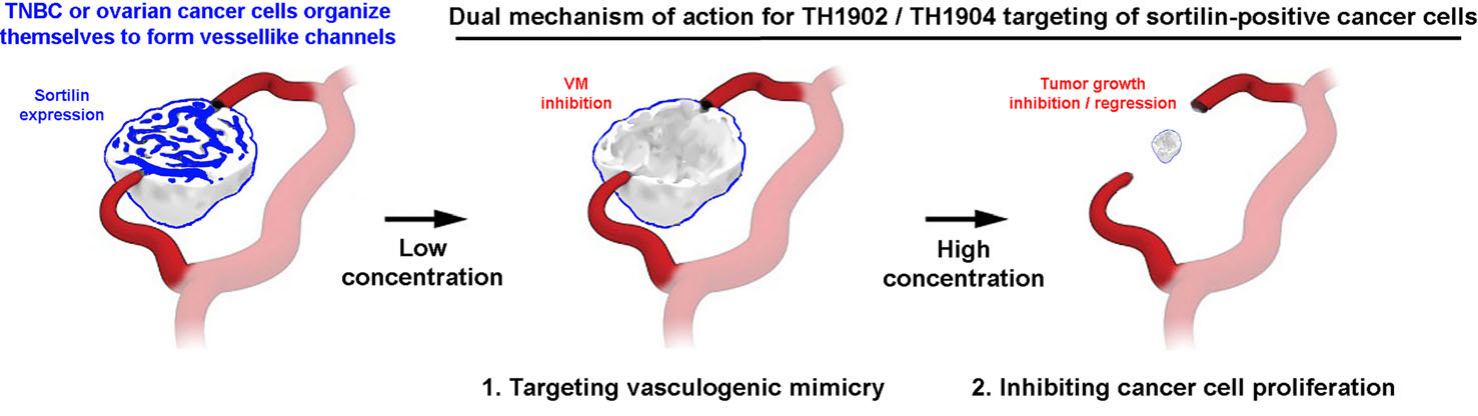
Dual mechanism of action for TH1902/TH1904 targeting of SORT1-positive cancers. VM (depicted in blue) is observed in SORT1-positive ovarian cancer and TNBC and can contribute to chemoresistance and metastasis by connecting to pre-existing blood vessels (depicted in red). A dual mechanism of action takes place with TH1902/TH1904 as, first, vasculogenic mimicry is inhibited at low nM concentrations and second, inhibition of cancer cell proliferation through the pharmacological action of the conjugated cytotoxic agent, namely Docetaxel/Doxorubucin, internalized through SORT1 functions. Altogether, this leads to precise and specific targeted inhibition of tumor growth (adapted from *Science 2016; 352(6292);1381-1383*, with the permission of the American Association for the Advancement of Science).

This study is the first to provide direct evidence that SORT1 is involved in the crucial molecular events required to generate VM in SORT1-positive ES-2 ovarian cancer and MDA-MB-231-derived TNBC cell models. Although acting as a key player in such process in these cell lines, this does not preclude the possible involvement of other molecular partners. A dual mechanism of action for TH1902/TH1904 targeting of SORT1-positive cancers is therefore hypothesized to take place (**Figure 10**). In fact, these are the first results indicating that in addition to their effects on SORT1-positive cancer cells, both conjugates also inhibited VM at low concentrations. Such pharmacological action of these peptide-drug conjugates is performed upon SORT1-mediated processes. Recently reported results suggest that stem-like CD133-positive human breast cancer cells can initiate *in vitro* VM (14, 15). Such evidence is in line with the current results which show a rapid post-transcriptional upregulation of CSC marker CD133 as well as an increase in MMP-9 expression. Hence, one could infer that, *in vivo*, VM may require SORT1 to interact with its tumor microenvironment and to also involve CSC. In addition, VM is significantly associated with larger tumor size and lymph node metastasis in breast cancer (41) and, given that some anti-angiogenesis therapies appear ineffective to block blood supply to tumor cells (53), inhibiting VM could become a beneficial alternative approach. Thus, the demonstrated inhibitory properties of TH1902 and TH1904 against VM, two novel peptide-drug conjugates that efficiently exploited SORT1-mediated internalization functions, further indicate that these conjugates have distinct anti-cancer properties from their unconjugated parental drugs, which could allow circumventing cancer resistance mechanisms.

## Data Availability Statement

The raw data supporting the conclusions of this article will be made available by the authors, without undue reservation.

## Ethics Statement

The animal study was reviewed and approved by Institutional Animal Care and Use Committee of Université du Québec à Montréal.

## Author Contributions

CC, MD, BA, and JCC carried out the design and coordination of the study. CC, MD, JCC, BD, AO, and AL performed *in vitro* experiments. AZ performed *in vivo* experiments. AL performed the synthesis of TH1902. CC, MD, J-CC, RB, CM, and BA analyzed the data and wrote the manuscript. All authors contributed to the article and approved the submitted version.

## Funding

This study was supported through fundings by the Programme de Soutien à la Valorisation et au Transfert (PSVT) from the Quebec government, and by the Canadian Cancer Society through the SynergiQc program from the Consortium Québécois sur la Découverte du Médicament (CQDM).

## Conflict of Interest

MD, AL, RB, and BA were scientific founders of Katana Biopharma. CM is senior vice president and chief medical officer at Theratechnologies. Authors CC, MD, JCC and AL was employed by Theratechnologies.

The remaining authors declare that the research was conducted in the absence of any commercial or financial relationships that could be construed as a potential conflict of interest.

## Publisher’s Note

All claims expressed in this article are solely those of the authors and do not necessarily represent those of their affiliated organizations, or those of the publisher, the editors and the reviewers. Any product that may be evaluated in this article, or claim that may be made by its manufacturer, is not guaranteed or endorsed by the publisher.

## Abbreviations

CSC, cancer stem cells; EC, endothelial cells; ECM, extracellular matrix; FAK, focal adhesion kinase; MMP, matrix metalloproteinases; NT, neurotensin; PAS, periodic acid-Schiff; SORT1, sortilin; TH1902, Docetaxel-TH19P01 conjugate; TH1904, Doxorubicin-TH19P01 conjugate; TH19P01, sortilin binding proprietary peptide; TNBC, triple negative breast cancer; VM, vasculogenic mimicry.
